# Objective disease activity assessment and therapeutic drug monitoring prior to biologic therapy changes in routine inflammatory bowel disease clinical practice: TARGET-IBD

**DOI:** 10.1186/s12876-022-02143-x

**Published:** 2022-02-19

**Authors:** Benjamin Click, Edward L. Barnes, Benjamin L. Cohen, Bruce E. Sands, John S. Hanson, David T. Rubin, Marla C. Dubinsky, Miguel Regueiro, Derek Gazis, Julie M. Crawford, Millie D. Long

**Affiliations:** 1 Department of Gastroenterology, Hepatology, and Nutrition, Cleveland Clinic, Cleveland, USA; 2 University of North Carolina, Chapel Hill, USA; 3 Mount Sinai, New York, USA; 4 Atrium Health, Charlotte, USA; 5 University of Chicago, Chicago, USA; 6 Target RWE Health Evidence Solutions, 2520 Meridian Pkwy, Suite 105, Durham, NC 27713 USA

**Keywords:** Real-world, Registry, Drug levels, Practice patterns, Disease monitoring

## Abstract

**Background:**

Inflammatory bowel disease (IBD) treatment paradigms recommend objective disease activity assessment and reactive therapeutic drug monitoring (TDM) prior to changes in biologic therapy. We aimed to describe objective marker and TDM assessment in routine clinical practice prior to biologic therapeutic changes in adult IBD patients.

**Methods:**

TARGET-IBD is a prospective longitudinal cohort of over 2100 IBD patients receiving usual care at 34 US academic or community centers enrolled between June 2017 and October 2019 who received biologic therapy and had a dose change or biologic discontinuation for lack of efficacy. Objective markers of disease activity within 12 weeks prior included fecal calprotectin, C-reactive protein (CRP), endoscopy, computed tomography (CT) and magnetic resonance imaging (MRI). TDM data for infliximab or adalimumab was obtained.

**Results:**

525 patients (71.4% Crohn’s disease [CD], 28.6% ulcerative colitis [UC]) receiving biologic therapy underwent dose change (55.6%) or discontinuation (44.4%) for lack of efficacy. The majority were Caucasian (85.7%), 18–39 years old (52.2%), privately insured (81.5%), and at academic centers (73.7%). For dose changes, 67.5% had at least one objective disease activity assessment or TDM in the 12 weeks prior (CD 67.9%, UC 66.2%; *P* = 0.79). The most common objective marker was CRP in both CD (39.1%) and UC (54.5%). CRP and calprotectin were used significantly more in UC (*P* = 0.02 and *P* = 0.03). TDM was obtained in 30.7% (28.8% UC, 31.4% CD; *P* = 0.72) prior to dose change. For biologic discontinuation, 79.4% patients underwent objective assessment or TDM prior. In CD, CRP (46.3%) was most common, and CT (*P* = 0.03) and MRI (*P* < 0.001) were significantly more frequent than in UC. TDM was performed in 40.1% of patients (43.5% UC, 38.0% CD, *P* = 0.49) prior to discontinuation. Among all participants with dose change or discontinuation, endoscopy was performed in 29.3% with CD and 31.3% with UC. Academic care setting was associated with objective assessment before therapy change (OR 1.59, 95% CI 1.01–2.50).

**Conclusion:**

Nearly one-third of patients undergoing a biologic dose change or discontinuation do not undergo objective disease activity assessment or TDM. Assessment choice differs by disease. Future studies assessing the impact of such practices on long-term outcomes are needed.

**Supplementary Information:**

The online version contains supplementary material available at 10.1186/s12876-022-02143-x.

## Background

The therapeutic landscape in inflammatory bowel disease (IBD) including both Crohn’s disease (CD) and ulcerative colitis (UC) is rapidly changing. The number of approved agents and mechanisms of action is expanding. Despite such innovation and evolution, the response rates of medical therapy for IBD remains suboptimal. Initial response rates in both clinical trials and real-world cohorts approach 50% [1–3]. Even in individuals that do initially respond, subsequent secondary loss of response is common, occurring in approximately 30–40% [4].

There are various reasons for symptoms of active disease despite biologic therapy [5–7]. These can include inadequate drug exposure, antibodies directed against the monoclonal antibody, active inflammation despite adequate drug exposure, disease complications (e.g. stricture, fistula, abscess), and finally non-IBD mediated etiologies (e.g., bile salt diarrhea [8], small intestinal bacterial overgrowth [9], irritable bowel syndrome [10]). Thus, understanding the reason for active symptoms is critical to determine the next step in management. Historically, such situations were managed by empirically escalating biologic dosing or changing to alternative therapy. With an expanding therapeutic armamentarium, there is increasing temptation to switch therapies when loss of efficacy is encountered. However, as more is now understood about mechanisms of disease breakthrough and symptom etiology, it is currently recommended that when faced with symptoms of active disease, clinicians should first discern whether the symptoms are due to active inflammation through objective assessments (e.g., endoscopy, cross- sectional imaging, biomarkers) and then perform therapeutic drug monitoring of the biologic to ascertain pharmacokinetic data [11–13]. Retrospective studies evaluating the efficacy of this approach have demonstrated cost-effectiveness and reductions in IBD-related complications [14–16]. Furthermore, both proactive routine objective monitoring for disease activity and therapeutic drug monitoring with resultant therapeutic adjustments have growing evidence bodies, with results demonstrating improved short- and long-term outcomes in IBD [14–16].

However, the real-world implementation of research findings and guideline recommendations often lags significantly. The aim of this study was to assess rates and influencing factors of objective disease activity assessment and therapeutic monitoring in IBD patients undergoing a change in their therapeutic regimen.

## Methods

### Data source

We performed an assessment of the TARGET-IBD cohort—a prospective longitudinal cohort of consented IBD patients receiving usual care from 34 academic and community centers throughout the US [17–19]. Adult and pediatric patients are eligible for TARGET-IBD if they carry a diagnosis of IBD and receive at least one prescription treatment. Diagnoses of IBD are made by standard of care assessments by the treating physician. Exclusion criteria include patients unable to provide written informed consent/assent, enrolled in any interventional study for IBD therapy, those with a history of prior total abdominal colectomy for UC, or those not meeting the inclusion criteria. As part of TARGET-IBD, participants have three years of redacted retrospective electronic medical records (EMR) submitted for centralized data abstraction by independent, trained employees of TARGET RWE with ongoing quality assurance and integrity monitoring. Submitted data includes clinic notes, endoscopy, radiography, pathology, and laboratory results, medication prescriptions, and health care utilization encounters. After enrollment, updated prospective interval medical records are submitted every 6 months for a total of 5 years prospective collection. Data obtained from outside vendors, labs, or facilities is submitted, abstracted, and included in the TARGET-IBD dataset when the information is available within the enrolling site’s EMR (e.g. scanned, documented, or manually input). Data from external facilities that is not integrated into the enrolling site’s EMR is not included.

### Study population

Adult (≥ 18 years) IBD participants who enrolled in TARGET-IBD between June 2017 (study launch) and October 2019, who were receiving biologic therapy during either the retrospective (three years prior to enrollment) or prospective periods following enrollment with a monoclonal antibody to treat IBD (infliximab, adalimumab, golimumab, certolizumab pegol, vedolizumab, or ustekinumab), and had either a dose escalation or biologic discontinuation were eligible for inclusion. For dose changes, only those with a centrally abstracted reason for therapeutic change identified as lack of efficacy were included. Lack of efficacy was defined by central abstraction as “no positive benefit for the patient” when interpreting clinical documentation. For discontinuations, those with identified reasons of lack of efficacy, antidrug antibodies, primary non-response, or secondary loss of response were included. Other reasons such as adverse event, non-medical switching, patient decision (including poor adherence), dose changes due to induction to maintenance transition, or unknown/other were excluded. Determinations were made by trained TARGET-IBD central data abstractors. Participants can have more than one reason identified. Sensitivity analysis of the reason for loss of efficacy was performed to ascertain impact of various clinical scenarios. Only the first therapeutic change in the study period was assessed. Participants who underwent surgery within 12 weeks were excluded.

### Outcomes

The outcomes of interest for this analysis were rates of objective disease activity assessment and therapeutic drug monitoring (TDM) in the 12 weeks prior to the date of therapeutic change. The date of change or discontinuation was enumerated as the date on which the prescription was altered for either a dose escalation or discontinuation. If the date of a change in therapy was not mentioned in the medical record, then the last date that the medication was listed in any part of the medical documentation was used. Objective disease activity assessment was defined by a resulted order for a surrogate biomarker (fecal calprotectin [FC] or C-reactive protein [CRP]), endoscopy (regardless of the specific endoscopic procedure or pre-existing disease location), or cross-sectional imaging of the abdomen and pelvis (including both computed tomography and magnetic resonance imaging). TDM data was limited to those receiving infliximab or adalimumab, due to the practical availability of TDM assays for these two agents during the study periods.

### Statistical analysis

Categorical variables were described as proportions and compared using Chi-square testing. Continuous variables were described using medians and ranges and compared using Student’s t-test or Mann Whitney U test where appropriate. The denominator for determining the proportion of the study population completing objective disease activity assessment included all study participants meeting eligibility criteria. For this analysis, we distinguished participants who underwent dose escalation from biologic discontinuation and evaluated each intervention individually. The denominator for evaluating rates of TDM performance was limited to those receiving infliximab or adalimumab.

We used multivariable logistic regression to evaluate factors associated with objective assessment or TDM including patient demographics, disease type (CD, UC), drug class, previous biologic use (yes/no), therapy change (discontinuation, dose change), disease duration at therapy change (years), insurance type, and site type (academic, community). We constructed separate models for objective assessments and TDM.

Sensitivity analyses were performed to assess the impact of time window definitions for objective assessment or TDM evaluation. These included extending the 12 week period to 24 weeks and also assessing 12 weeks after dose escalation or change.

All statistical tests were two-sided with alpha = 0.05 unless otherwise mentioned. All analyses were carried out using SAS (version 9.4) statistical software (SAS Institute, Cary, NC, USA).

## Results

### Study population

**Fig. 1:**
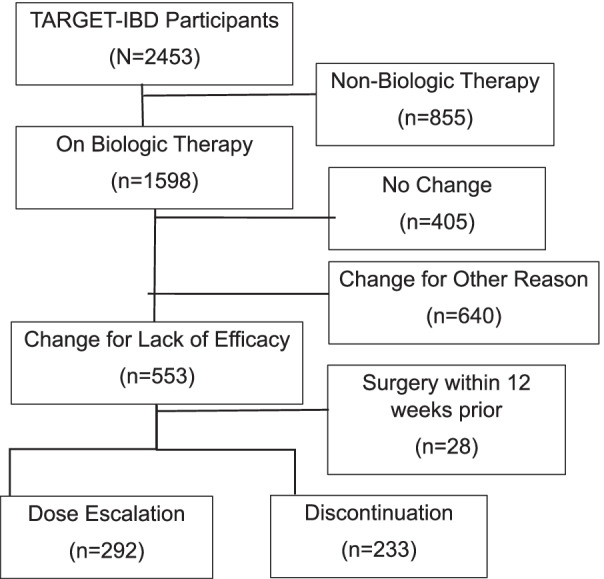
Study population flow diagram from TARGET-IBD consented cohort

As of October 2019, 2453 participants were enrolled in TARGET-IBD, of whom 1598 (65.1%) received a biologic therapy. A total of 525 participants (71.4% CD, 28.6% UC) underwent either a dose change (55.6%) or biologic discontinuation (44.4%) during the study period and formed the study population (Fig. 1). The majority were female (58.3%), Caucasian (85.7%), between 18 and 39 years old (51.8%), privately insured (81.5%) and enrolled at an academic center (73.7%) (Table 1). In CD, there was approximately 1/3 with inflammatory (35.5%), stricturing (29.9%), or penetrating (34.7%) disease as well as 30.7% with perianal involvement. In UC participants, the majority (66.7%) had extensive colitis. Adalimumab (43.8%) was the most commonly used biologic, followed by infliximab (30.5%).

**Table 1:** Baseline demographics, disease characteristics, medication history and utilization of study population

Variable	All participants (N = 525)	Participant type	
		Ulcerative colitis (N = 150)	Crohn's disease (N = 375)
Age at study entry (years)			
Median (n)	39 (525)	39 (150)	39 (375)
Min–max	18–80	19–74	18–80
Age at study entry by category, n (%)^1^			
18–39	274 (52.2%)	78 (52.0%)	196 (52.3%)
40–64	199 (37.9%)	53 (35.3%)	146 (38.9%)
≥ 65	52 (9.9%)	19 (12.7%)	33 (8.8%)
Age at diagnosis (years)			
Median (n)	26 (494)	32 (144)	24 (350)
Min–max	1–71	1–71	6–66
Age at diagnosis by category, n (%)			
< 18	106 (20.2%)	18 (12.0%)	88 (23.5%)
18–39	272 (51.8%)	75 (50.0%)	197 (52.5%)
40–49	56 (10.7%)	22 (14.7%)	34 (9.1%)
50–64	52 (9.9%)	23 (15.3%)	29 (7.7%)
≥ 65	8 (1.5%)	6 (4.0%)	2 (0.5%)
Not reported	31 (5.9%)	6 (4.0%)	25 (6.7%)
Sex, n (%)			
Female	306 (58.3%)	83 (55.3%)	223 (59.5%)
Male	219 (41.7%)	67 (44.7%)	152 (40.5%)
Race, n (%)			
White	450 (85.7%)	128 (85.3%)	322 (85.9%)
Black or African American	36 (6.9%)	9 (6.0%)	27 (7.2%)
Native Hawaiian or Other Pacific Islander	2 (0.4%)	1 (0.7%)	1 (0.3%)
Asian	6 (1.1%)	1 (0.7%)	5 (1.3%)
Other	12 (2.3%)	4 (2.7%)	8 (2.1%)
Not reported	19 (3.6%)	7 (4.7%)	12 (3.2%)
Ethnicity, n (%)			
Hispanic or Latino	20 (3.8%)	4 (2.7%)	16 (4.3%)
Not Hispanic or Latino	478 (91.0%)	134 (89.3%)	344 (91.7%)
Other	2 (0.4%)	1 (0.7%)	1 (0.3%)
Not reported	25 (4.8%)	11 (7.3%)	14 (3.7%)
Insurance type at enrollment	525 (100.0%)	150 (100.0%)	375 (100.0%)
Private	428 (81.5%)	122 (81.3%)	306 (81.6%)
Medicare	59 (11.2%)	19 (12.7%)	40 (10.7%)
Medicaid	42 (8.0%)	14 (9.3%)	28 (7.5%)
Supplemental	17 (3.2%)	5 (3.3%)	12 (3.2%)
Other	8 (1.5%)	2 (1.3%)	6 (1.6%)
Unknown	7 (1.3%)	1 (0.7%)	6 (1.6%)
Site type, n (%)			
Academic	387 (73.7%)	101 (67.3%)	286 (76.3%)
Community	138 (26.3%)	49 (32.7%)	89 (23.7%)
Crohn's disease location, n (%)			
n	375	–	375
Colon	52 (13.9%)		52 (13.9%)
Ileocolon	226 (60.3%)		226 (60.3%)
Ileum	92 (24.5%)		92 (24.5%)
Not reported	5 (1.3%)		5 (1.3%)
Upper GI tract involvement, n (%)^1^			
n	375	–	375
No	167 (44.5%)		167 (44.5%)
Yes	79 (21.1%)		79 (21.1%)
Not reported	129 (34.4%)		129 (34.4%)
Crohn's disease behavior, n (%)			
n	375	–	375
Inflammatory (B1)	133 (35.5%)		133 (35.5%)
Stricturing (B2)	112 (29.9%)		112 (29.9%)
Penetrating/fistulizing (B3)	130 (34.7%)		130 (34.7%)
Perianal disease, n (%)			
n	375	–	375
No	156 (41.6%)		156 (41.6%)
Yes	115 (30.7%)		115 (30.7%)
Not reported	104 (27.7%)		104 (27.7%)
Ulcerative colitis extent, n (%)			
n	150	150	–
Extensive	100 (66.7%)	100 (66.7%)	
Left-sided	40 (26.7%)	40 (26.7%)	
Proctitis	2 (1.3%)	2 (1.3%)	
Not reported	8 (5.3%)	8 (5.3%)	
Previous biologic use, n (%)^1^			
No	295 (56.2%)	116 (77.3%)	179 (47.7%)
Yes	230 (43.8%)	34 (22.7%)	196 (52.3%)
Biologic, n (%)			
Adalimumab	230 (43.8%)	69 (46.0%)	161 (42.9%)
Certolizumab	34 (6.5%)	2 (1.3%)	32 (8.5%)
Etanercept	1 (0.2%)	0 (0.0%)	1 (0.3%)
Golimumab	3 (0.6%)	1 (0.7%)	2 (0.5%)
Infliximab	160 (30.5%)	52 (34.7%)	108 (28.8%)
Ustekinumab	24 (4.6%)	0 (0.0%)	24 (6.4%)
Vedolizumab	73 (13.9%)	26 (17.3%)	47 (12.5%)
Objective assessment or TDM, n (%)			
No assessment prior to treatment change	143 (27.2%)	41 (27.3%)	102 (27.2%)
Objective assessment only	245 (46.7%)	65 (43.3%)	180 (48.0%)
TDM only	32 (6.1%)	11 (7.3%)	21 (5.6%)
Objective assessment and TDM	105 (20.0%)	33 (22.0%)	72 (19.2%)

^1^Includes biologic use prior to the initiation of the biologic for which there was a dose change or discontinuation due to lack of effect

### Biologic dose escalation outcomes

**Fig. 2:**
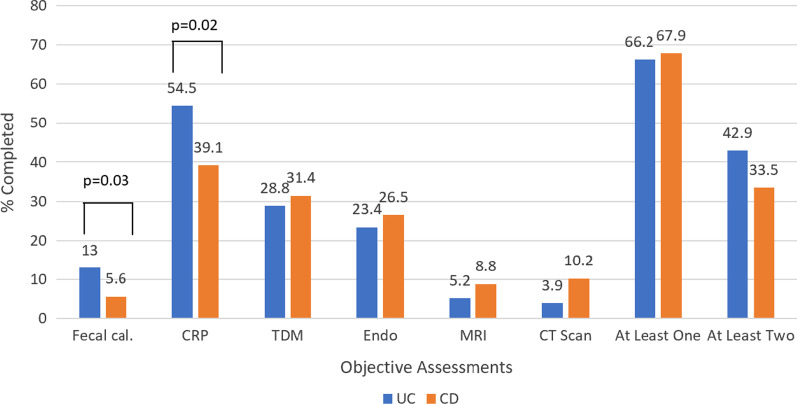
Rates of objective disease activity assessment and therapeutic drug monitoring (TDM) prior to dose escalation of biologic therapy. (N = 292; 77 with UC and 215 with CD)

Of patients who underwent a dose escalation (n = 292, 55.6% of study population), 67.5% had at least one objective disease activity assessment or TDM in the 12 weeks prior and 36.0% had two or more (Fig. 2). These rates did not significantly differ by disease (*P* = 0.79 for one or more; *P* = 0.14 for two or more assessments). The most common objective marker utilized was CRP in both CD (39.1%) and UC (54.5%), whereas FC was utilized relatively infrequently (CD 5.6%, UC 13.0%). Endoscopy was performed in about ¼ participants (26.5% CD, 23.4% UC) and cross-sectional imaging was uncommon (CT 8.6%, MRI 7.9%). Of those participants who underwent a colonoscopy (CD n = 45, UC n = 9), 83.3% had an abnormal result (CD 80.0%, UC 100%). Comparing diseases, CRP and FC were used significantly more in UC than CD (*P* = 0.02 and 0.03, respectively) while CT was utilized more in CD though this did not reach significance (*P* = 0.09).

Of participants receiving infliximab (36.3%) or adalimumab (41.8%), 28.8% of UC subjects and 31.4% of CD subjects undergoing dose escalation had TDM performed prior to this therapeutic change. This did not differ by disease (*P* = 0.72). Rates of TDM testing were not significantly different by biologic agent (adalimumab 34.4% and infliximab 26.4%, *P* = 0.19).

### Biologic discontinuation outcomes

**Fig. 3:**
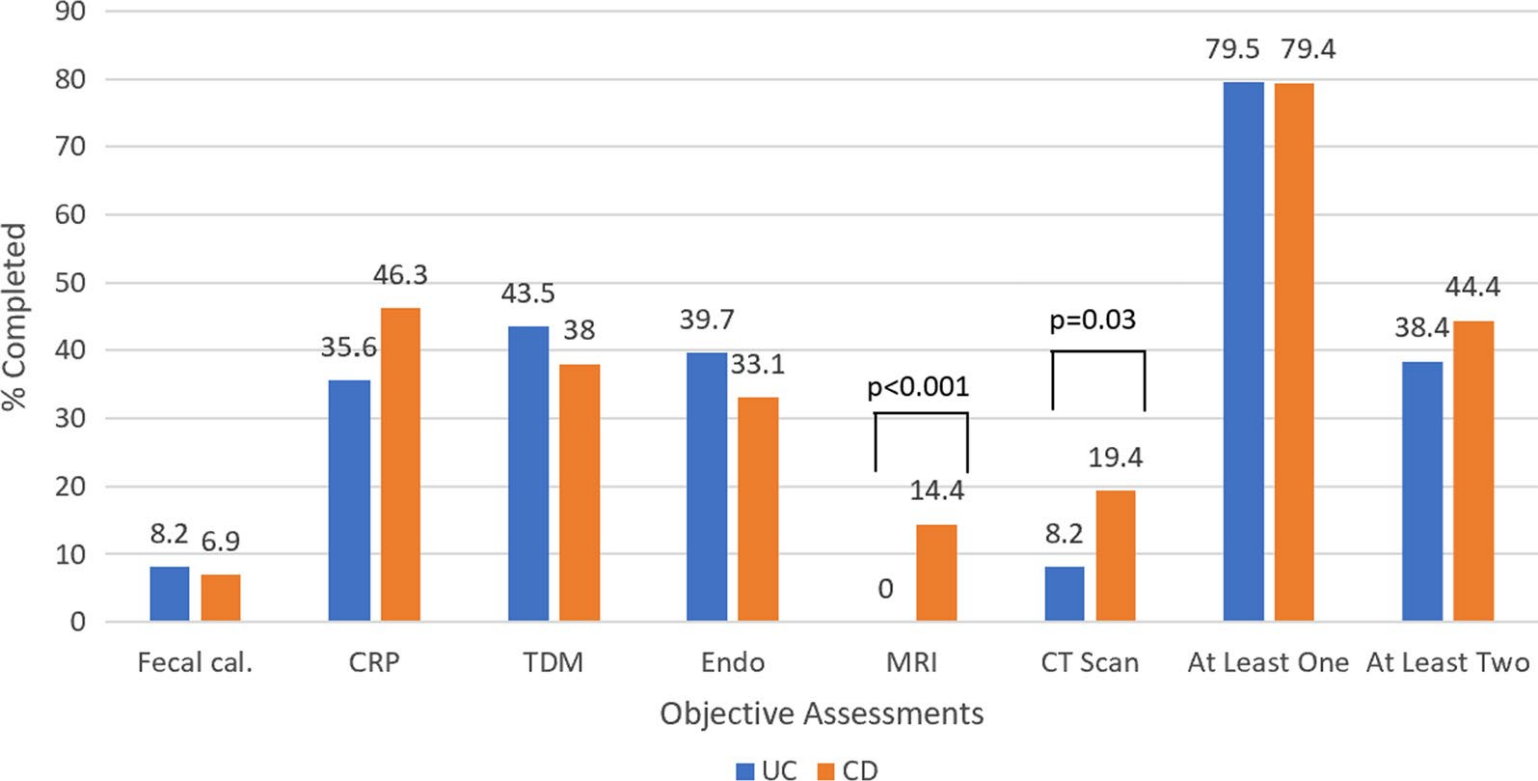
Rates of objective disease activity assessment and therapeutic drug monitoring (TDM) prior to dose escalation of biologic therapy. (N = 233; 73 with UC and 160 with CD)

Of patients who had a biologic discontinuation (n = 233, 44.4% of study population), 79.4% had at least one objective assessment or TDM performed in the 12 weeks prior, and 42.5% had two or more (Fig. 3). The most frequently utilized measure was CRP in CD (46.3%) and endoscopy in UC (39.7%). Similar to dose escalation, FC was infrequently utilized (6.9% CD, 8.2% UC). CD participants underwent significantly more cross-sectional imaging compared to UC, (CT 19.4% vs. 8.2%, respectively, *P* = 0.03; MRI 14.4% vs. 0%, *P* < 0.001).

TDM was performed in 38.0% CD participants and 43.5% of UC subjects receiving infliximab or adalimumab (*P* = 0.49). Prior to discontinuation, TDM was performed significantly more frequently in infliximab (51.9%) treated participants than adalimumab (34.3%, *P* = 0.03).

### Factors associated with objective assessment or TDM

Multiple logistic regression modeling of included variables did not identify any patient or disease factors significantly associated with TDM measurement (Table 2). Age, sex, race, previous biologic use, disease type (CD vs. UC) disease duration, insurance status, or therapy change (discontinuation vs. dose change) were not associated with receiving objective assessment. Patients treated at academic centers were more likely to have objective assessment than those receiving care at community sites (OR 1.59, 95% CI 1.01–2.50), and patients on infliximab (OR 0.57, 95% CI 0.36–0.90) and ustekinumab (OR 0.35, 95% CI 0.14–0.91) were less likely to have objective assessment compared to those on adalimumab.

**Table 2:** Multivariable regression models for objective assessment and TDM performance in the 12 weeks prior to therapy change (either dose change or discontinuation)

	At least one objective assessment^a^			TDM performed		
	OR	95% CI	*p* value	OR	95% CI	*p* value
Sex (female vs. male)	0.849	0.565–1.274	0.4283	1.480	0.929–2.361	0.0993
Race (white vs. nonwhite)	0.657	0.330–1.306	0.2305	1.152	0.540–2.456	0.7147
Age at therapy change (years)	1.004	0.989–1.019	0.6215	0.996	0.979–1.013	0.6164
Previous biologic use (yes vs. no)	1.027	0.639–1.652	0.9120	0.980	0.5840–1.642	0.9378
Disease duration at therapy change (years)	0.987	0.964–1.011	0.2896	0.997	0.969–1.025	0.8133
Site type (academic vs. community)	1.588	1.010–2.495	0.0452	1.114	0.662–1.875	0.6845
Private insurance at enrollment (yes vs. no)	1.044	0.602–1.811	0.8781	1.188	0.626–2.253	0.5979
Drug class^b^			0.0083			
Adalimumab	Ref	Ref	–	Ref	Ref	Ref
Infliximab	0.569	0.360–0.900	0.0159	0.844	0.529–1.348	0.4779
Other anti-TNF	1.460	0.576–3.705	0.4252			
Ustekinumab	0.353	0.137–0.914	0.0320			
Vedolizumab	1.359	0.683–2.702	0.3819			
Disease type (CD vs. UC)	1.181	0.734–1.901	0.4923	0.953	0.567–1.602	0.8566
Therapy change (discontinuation vs. dose change)	1.178	0.776–1.788	0.4417	1.011	0.634–1.613	0.9632

^a^Objective assessment included fecal calprotectin, C-reactive protein, endoscopy, MRI, or CT scan

^b^For TDM model, drug class compared infliximab to adalimumab (reference) as these were the only two biologics included in the TDM assessment population

### Sensitivity analyses

In order to assess the impact of the chosen time horizon of objective assessment or TDM, we performed a sensitivity analysis extending the time window to 6 months before therapy alteration (Additional file 1: Tables S1a, S1b). The increase from 3 to 6 months showed that an additional 10.9% of participants had objective assessment or TDM prior to dose change (78.4% all; 81.8% UC; 77.2% CD) (Additional file 1: Table S1a). Similarly, in participants who discontinued therapy, extending the assessment interval to 6 months demonstrated an increase of 7.7% undergoing objective assessment or TDM in the 6 months prior to discontinuation (87.1% all; 84.9% UC; 88.1% CD) (Additional file 1: Table S1b). Due to practice variation (e.g. ordering objective assessments while simultaneously coordinating therapeutic changes), we also performed an analysis extending the time window from 3 months before to 3 months after the therapy change (Additional file 1: Tables S2a, S2b). This demonstrated an additional 10.2% of participants had objective assessment or TDM in the 3 months after dose change (77.7% all; 76.6% UC; 78.1% CD) (Additional file 1: Table S2a), and an additional 9.4% were objectively assessed in the 3 months after therapy discontinuation (88.8% all; 86.3% UC; 90.0% CD) (Additional file 1: Table S2b).

## Discussion

In this cohort study of IBD patients receiving biologic therapy and undergoing treatment changes, up to 1/3 of individuals did not undergo objective disease activity assessment or therapeutic drug monitoring prior to the modification. In patients receiving biologics with routinely available TDM capability, nearly 2/3 of subjects did not undergo TDM evaluation. Understanding reasons and mechanisms for clinical loss of response are imperative to therapeutic decision making to optimize outcomes in IBD patients on biologics.

Societal guideline statements currently recommend assessing for objective disease activity when faced with new or recurrent symptoms and performing reactive TDM in light of secondary loss of response [12]. The current study suggests that while at least one objective assessment occurs in the majority of individuals prior to a change or discontinuation of a biologic therapy for lack of clinical efficacy, a significant portion do not. Even extending the assessment window to 6 months before or including the 3-month period after a dose adjustment or therapy discontinuation only yielded an additional 7 to 10% participants undergoing objective evaluation or TDM (Additional file 1: Tables S1a–S2b). This suggests that some clinicians are relying on clinical symptomatology and empiric therapeutic adjustments rather than objective data. Potential alternative explanations for the lack of universal assessment include: (1) testing performed at outside centers; (2) other objective metrics utilized such as erythrocyte sedimentation rate or fecal lactoferrin, (3) local health care or payer environment results in limited ability to utilize these assessments universally in routine clinical practice, or (4) certain clinical situations dictate other action (e.g., ongoing disease flare > 3–6 months, severely ill patient). It should be noted that if the results are available in the enrolling center’s EMR (e.g., scanned lab results), it was included in the TARGET-IBD data; however, data from other institutions via interoperability platforms (e.g. Care Everywhere in Epic) is not transmitted or abstracted by TARGET-IBD due to legal restrictions. While only CRP and FC were assessed in this study, these markers have demonstrated superior test characteristics compared to other biomarkers [20–22]. Another potential explanation includes delayed adaptation of such assessment strategies due to the body of evidence available at the time and clinician discretion in implementing the recommended strategies.

On multivariable modeling, the completion of objective assessment prior to a change in therapy was independently associated with site of care, with academic centers being 59% more likely to perform than community centers. There are several possible explanations, including more familiarity with recently published studies or guidelines in academic centers, clinical workload or support differences between sites of care to obtain and interpret such studies, or variable access to some of the markers such as calprotectin or MRI. Reasons for the differences between sites of care should be explored in future studies and efforts made to bridge any gaps or opportunities identified.

Interestingly, compared to participants receiving adalimumab, those receiving infliximab were significantly less likely to undergo objective assessment before a change in therapy. The exact reason for this difference is unclear. Potential explanations include the wide dosing range available to infliximab, familiarity with alternative dosing strategies, or differences in payer coverage or requirements for dose changes between the two agents. Furthermore, those receiving ustekinumab were also less likely to undergo objective assessments compared to adalimumab. We hypothesize this may be due to utilization of ustekinumab in a more biologic refractory population during the study period as well as more nascent data on TDM implications with ustekinumab compared to anti-TNFs. Thus, when faced with symptoms suggestive of active disease, a dose change may be implemented empirically as limited medical alternatives existed. These biologic specific differences were not seen in the TDM model. Additional studies are needed to explore these differences and potential reasons.

In the current study, nearly 2/3 of individuals receiving infliximab or adalimumab did not undergo TDM prior to a therapeutic change, either dose or agent. The utility of TDM in aiding therapeutic decision making and influencing disease-related costs and outcomes has been demonstrated in several retrospective and prospective studies [16, 23–28]. The current data suggests that real-world utilization of TDM, despite American Gastroenterological Association recommendations [12], still faces significant practice implementation challenges. These may include provider (e.g., lack of knowledge about TDM, critical position on TDM, lack of access or difficulty with ordering process), payer (e.g., lack of coverage, large out of pocket expenses), patient (e.g., does not obtain testing even when ordered, distance to travel), or system (e.g., lack of in-house testing) explanations. In a survey of gastroenterologists, lack of insurance coverage and high out of pocket costs were cited by a majority of responders as significant barriers to TDM implementation in real-world practice [29]. Our data reflects these challenges despite limiting the TDM patient population to those receiving infliximab or adalimumab, the two agents with most available TDM data and commercial testing ability. Thus, there remains a significant discordance between data-driven guideline recommendations and real-world capability and practice patterns.

With an increasing breadth of biologic and small molecule options for treating IBD, there may be a temptation by clinicians to simply change biologic therapy rather than assess and optimize when faced with potential loss of efficacy. However, the number of available treatments is not infinite. Multiple studies have demonstrated that subsequent biologic agents are typically less efficacious than the first and often more costly. Thus, emphasis must be laid on understanding and optimizing available therapies. Furthermore, in the pivotal CALM trial [15], proactive objective assessments of biomarkers and disease activity with resultant changes in the therapy when indicated improved Crohn’s disease outcomes. Despite this, real-world objective assessments after initiating biologic therapy remains incomplete. In a study of practice patterns from 2007 to 2016 using the Truven MarketScan database, Limketkai et al., reported 56.4% of CD patients and 67.8% of UC patients undergoing objective evaluations within 6 months of starting a biologic, rates similar to the current reactive evaluation [30]. The authors also demonstrated geographic variability, substantiating the inconsistency in real-world practice patterns. Accordingly, there remains a gap in real-world disease evaluation and biologic optimization that may influence IBD population-level outcomes.

Limitations of this study include the utilization of available clinical information. Certain data such as outside testing or procedures could be missing if not incorporated into the enrolling investigator’s EMR. However, for TDM data, which can include outside lab testing facilities, if testing was performed and mentioned in the clinical documentation, this was counted as performed, even if results not available. These limitations are similar to almost all real-world studies relying upon available clinical documentation and data. The majority of the study population was enrolled by an academic center, and thus may create both referral bias and generalizability issues. To this end, if the majority of patients are seen at academic centers and standard of care is deviating from guideline recommendations, this is even more critical to understand and reflective of real-world practice patterns. Similarly, TARGET-IBD is derived from U.S. centers and generalizability to other health care systems is limited. The exact reason for lack of testing on an individual level is unknown; however, we included only those with reasons for change or discontinuation as lack of efficacy, nonresponse, or antidrug antibody formation. Such a population represents scenarios where objective testing or TDM is most likely to be pursued. However, due to lack of prospective determination of reasons for change, full generalizability to all clinical situations is limited. Data limitations of TARGET-IBD include lack of detailed socioeconomic status data that may influence access to specialty care and related testing. In this study, only patients on infliximab or adalimumab were included in the TDM analysis and thus rates of TDM for other biologics is unknown. This was chosen as these agents have the most robust TDM data and available commercial testing capability. Including all biologics would be valuable, as many of the second line therapies are expensive; moreover, this likely would have resulted in even lower rates of TDM assessment.

## Conclusions

In conclusion, in a large prospective IBD registry cohort, objective disease activity assessment and therapeutic drug monitoring prior to changes in biologic regimen were not performed in a substantial portion of participants. Improved understanding of the reasons for practice variability and the long-term impacts of these practice patterns may help optimize biologic utilization and disease outcomes for IBD patients.

## Supplementary Information

**Additional file 1: Table 1a**. TARGET-IBD Labs/Imaging 24 Weeks Prior to Dose Changes of Biologic Therapy Due to Lack of Effect.** Table 1b**. TARGET-IBD Labs/Imaging 24 Weeks Prior to Discontinuations of Biologic Therapy Due to Lack of Effect.** Table 2a**. TARGET-IBD Labs/Imaging within 12 Weeks Before or After Dose Changes of Biologic Therapy Due to Lack of Effect.** Table 2b**. TARGET-IBD Labs/Imaging within 12 Weeks Before or After Discontinuations of Biologic Therapy Due to Lack of Effect.

## Data Availability

The authors declare that the data supporting the findings of this study are available within the article and its Additional information files.

## Abbreviations

IBD: Inflammatory bowel disease
TDM: Therapeutic drug monitoring
CRP: C-reactive protein
CT: Computed tomography
MRI: Magnetic resonance imaging
CD: Crohn’s disease
UC: Ulcerative colitis
EMR: Electronic medical records
FC: Fecal calprotectin
