# Supplementary material for: Objective disease activity assessment and therapeutic drug monitoring prior to biologic therapy changes in routine inflammatory bowel disease clinical practice: TARGET-IBD
Source: BMC Gastroenterol. 2022 Feb 19;22:71. doi: 10.1186/s12876-022-02143-x (PMC8858477; doi:10.1186/s12876-022-02143-x)
Supplement: Supplementary file 1 — Additional file 1: Table 1a. TARGET-IBD Labs/Imaging 24 Weeks Prior to Dose Changes of Biologic Therapy Due to Lack of Effect. Table 1b. TARGET-IBD Labs/Imaging 24 Weeks Prior to Discontinuations of Biologic Therapy Due to Lack of Effect. Table 2a. TARGET-IBD Labs/Imaging within 12 Weeks Before or After Dose Changes of Biologic Therapy Due to Lack of Effect. Table 2b. TARGET-IBD Labs/Imaging within 12 Weeks Before or After Discontinuations of Biologic Therapy Due to Lack of Effect. [file 12876_2022_2143_MOESM1_ESM.docx]

| Supplementary Table 1a. TARGET-IBD Labs/Imaging 24 Weeks Prior to Dose Changes of Biologic Therapy Due to Lack of Effect | | | | |  |
| --- | --- | --- | --- | --- | --- |
| **Lab/Imaging Done Within 24 Weeks Prior to Dose Change?, n (%)** | **All Participants (N=292)** | **Ulcerative Colitis (N=77)** | **Crohn's Disease (N=215)** | **P-value^1^** | |
| Fecal Calprotectin   No   Yes | 260 (89.0%)  32 (11.0%) | 64 (83.1%)  13 (16.9%) | 196 (91.2%)  19 (8.8%) | 0.0529 | |
| C-Reactive Protein   No   Yes | 131 (44.9%)  161 (55.1%) | 27 (35.1%)  50 (64.9%) | 104 (48.4%)  111 (51.6%) | 0.0443 | |
| Therapeutic Drug Monitoring^2^  n   No   Yes | 228  145 (63.6%)  83 (36.4%) | 59  41 (69.5%)  18 (30.5%) | 169  104 (61.5%)  65 (38.5%) | 0.2754 | |
| Endoscopy   No   Yes | 167 (57.2%)  125 (42.8%) | 38 (49.4%)  39 (50.6%) | 129 (60.0%)  86 (40.0%) | 0.1057 | |
| MRI   No   Yes | 256 (87.7%)  36 (12.3%) | 71 (92.2%)  6 (7.8%) | 185 (86.0%)  30 (14.0%) | 0.1589 | |
| CT Scan   No   Yes | 251 (86.0%)  41 (14.0%) | 70 (90.9%)  7 (9.1%) | 181 (84.2%)  34 (15.8%) | 0.1458 | |
| At Least One of the Above   No   Yes | 63 (21.6%)  229 (78.4%) | 14 (18.2%)  63 (81.8%) | 49 (22.8%)  166 (77.2%) | 0.3997 | |
| At Least Two of the Above   No   Yes | 137 (46.9%)  155 (53.1%) | 32 (41.6%)  45 (58.4%) | 105 (48.8%)  110 (51.2%) | 0.2729 | |
| ^1^ P-values are from general association tests for difference in objective disease activity assessment by disease type. | | | | |  |
| ^2^ Therapeutic drug monitoring includes antibodies or serum concentration labs for adalimumab or infliximab. | | | | |  |
| Note: This table includes participants whose first recorded therapeutic change of a biologic due to lack of effect is a dose change, not a discontinuation. Only the first such change is analyzed. Participants who had a surgery within 12 weeks prior to this change are excluded. | | | | |  |

| Supplementary Table 1b. TARGET-IBD Labs/Imaging 24 Weeks Prior to Discontinuations of Biologic Therapy Due to Lack of Effect | | | | |  |
| --- | --- | --- | --- | --- | --- |
| **Lab/Imaging Done Within 24 Weeks Prior to Discontinuation?, n (%)** | **All Participants (N=233)** | **Ulcerative Colitis (N=73)** | **Crohn's Disease (N=160)** | **P-value^1^** | |
| Fecal Calprotectin   No   Yes | 206 (88.4%)  27 (11.6%) | 63 (86.3%)  10 (13.7%) | 143 (89.4%)  17 (10.6%) | 0.4975 | |
| C-Reactive Protein   No   Yes | 112 (48.1%)  121 (51.9%) | 41 (56.2%)  32 (43.8%) | 71 (44.4%)  89 (55.6%) | 0.0955 | |
| Therapeutic Drug Monitoring^2^  n   No   Yes | 162  89 (54.9%)  73 (45.1%) | 62  30 (48.4%)  32 (51.6%) | 100  59 (59.0%)  41 (41.0%) | 0.1883 | |
| Endoscopy   No   Yes | 123 (52.8%)  110 (47.2%) | 37 (50.7%)  36 (49.3%) | 86 (53.8%)  74 (46.3%) | 0.6645 | |
| MRI   No   Yes | 202 (86.7%)  31 (13.3%) | 73 (100.0%)  0 (0.0%) | 129 (80.6%)  31 (19.4%) | <.0001 | |
| CT Scan   No   Yes | 176 (75.5%)  57 (24.5%) | 64 (87.7%)  9 (12.3%) | 112 (70.0%)  48 (30.0%) | 0.0037 | |
| At Least One of the Above   No   Yes | 30 (12.9%)  203 (87.1%) | 11 (15.1%)  62 (84.9%) | 19 (11.9%)  141 (88.1%) | 0.5005 | |
| At Least Two of the Above   No   Yes | 92 (39.5%)  141 (60.5%) | 34 (46.6%)  39 (53.4%) | 58 (36.3%)  102 (63.8%) | 0.1356 | |
| ^1^ P-values are from general association tests for difference in objective disease activity assessment by disease type. | | | | |  |
| ^2^ Therapeutic drug monitoring includes antibodies or serum concentration labs for adalimumab or infliximab. Therapeutic drug monitoring is also considered to have occurred if the reason given for treatment discontinuation is Antibodies Developed, even if there is no lab data available. | | | | |  |
| Note: This table includes participants whose first recorded therapeutic change of a biologic due to lack of effect is a discontinuation, not a dose change. Only the first such change is analyzed. Participants who had a surgery within 12 weeks prior to this change are excluded. | | | | |  |

| Supplementary Table 2a. TARGET-IBD Labs/Imaging within 12 Weeks Before or After Dose Changes of Biologic Therapy Due to Lack of Effect | | | | |  |
| --- | --- | --- | --- | --- | --- |
| **Lab/Imaging Done Within 12 Weeks Before to 12 Weeks After Dose Change?, n (%)** | **All Participants (N=292)** | **Ulcerative Colitis (N=77)** | **Crohn's Disease (N=215)** | **P-value^1^** | |
| Fecal Calprotectin   No   Yes | 260 (89.0%)  32 (11.0%) | 64 (83.1%)  13 (16.9%) | 196 (91.2%)  19 (8.8%) | 0.0529 | |
| C-Reactive Protein   No   Yes | 135 (46.2%)  157 (53.8%) | 29 (37.7%)  48 (62.3%) | 106 (49.3%)  109 (50.7%) | 0.0793 | |
| Therapeutic Drug Monitoring^2^  n   No   Yes | 228  140 (61.4%)  88 (38.6%) | 59  34 (57.6%)  25 (42.4%) | 169  106 (62.7%)  63 (37.3%) | 0.4898 | |
| Endoscopy   No   Yes | 190 (65.1%)  102 (34.9%) | 51 (66.2%)  26 (33.8%) | 139 (64.7%)  76 (35.3%) | 0.8030 | |
| MRI   No   Yes | 252 (86.3%)  40 (13.7%) | 72 (93.5%)  5 (6.5%) | 180 (83.7%)  35 (16.3%) | 0.0324 | |
| CT Scan   No   Yes | 255 (87.3%)  37 (12.7%) | 70 (90.9%)  7 (9.1%) | 185 (86.0%)  30 (14.0%) | 0.2719 | |
| At Least One of the Above   No   Yes | 65 (22.3%)  227 (77.7%) | 18 (23.4%)  59 (76.6%) | 47 (21.9%)  168 (78.1%) | 0.7841 | |
| At Least Two of the Above   No   Yes | 146 (50.0%)  146 (50.0%) | 37 (48.1%)  40 (51.9%) | 109 (50.7%)  106 (49.3%) | 0.6908 | |
| ^1^ P-values are from general association tests for difference in objective disease activity assessment by disease type. | | | | |  |
| ^2^ Therapeutic drug monitoring includes antibodies or serum concentration labs for adalimumab or infliximab. | | | | |  |
| Note: This table includes participants whose first recorded therapeutic change of a biologic due to lack of effect is a dose change, not a discontinuation. Only the first such change is analyzed. Participants who had a surgery within 12 weeks prior to this change are excluded. | | | | |  |

| Supplementary Table 2b. TARGET-IBD Labs/Imaging within 12 Weeks Before or After Discontinuations of Biologic Therapy Due to Lack of Effect | | | | |  |
| --- | --- | --- | --- | --- | --- |
| **Lab/Imaging Done Within 12 Weeks Before to 12 Weeks After Discontinuation?, n (%)** | **All Participants (N=233)** | **Ulcerative Colitis (N=73)** | **Crohn's Disease (N=160)** | **P-value^1^** | |
| Fecal Calprotectin   No   Yes | 207 (88.8%)  26 (11.2%) | 63 (86.3%)  10 (13.7%) | 144 (90.0%)  16 (10.0%) | 0.4066 | |
| C-Reactive Protein   No   Yes | 103 (44.2%)  130 (55.8%) | 38 (52.1%)  35 (47.9%) | 65 (40.6%)  95 (59.4%) | 0.1040 | |
| Therapeutic Drug Monitoring^2^  n   No   Yes | 162  90 (55.6%)  72 (44.4%) | 62  32 (51.6%)  30 (48.4%) | 100  58 (58.0%)  42 (42.0%) | 0.4279 | |
| Endoscopy   No   Yes | 124 (53.2%)  109 (46.8%) | 34 (46.6%)  39 (53.4%) | 90 (56.3%)  70 (43.8%) | 0.1707 | |
| MRI   No   Yes | 193 (82.8%)  40 (17.2%) | 70 (95.9%)  3 (4.1%) | 123 (76.9%)  37 (23.1%) | 0.0004 | |
| CT Scan   No   Yes | 180 (77.3%)  53 (22.7%) | 64 (87.7%)  9 (12.3%) | 116 (72.5%)  44 (27.5%) | 0.0106 | |
| At Least One of the Above   No   Yes | 26 (11.2%)  207 (88.8%) | 10 (13.7%)  63 (86.3%) | 16 (10.0%)  144 (90.0%) | 0.4066 | |
| At Least Two of the Above   No   Yes | 90 (38.6%)  143 (61.4%) | 30 (41.1%)  43 (58.9%) | 60 (37.5%)  100 (62.5%) | 0.6018 | |
| ^1^ P-values are from general association tests for difference in objective disease activity assessment by disease type. | | | | |  |
| ^2^ Therapeutic drug monitoring includes antibodies or serum concentration labs for adalimumab or infliximab. Therapeutic drug monitoring is also considered to have occurred if the reason given for treatment discontinuation is Antibodies Developed, even if there is no lab data available. | | | | |  |
| Note: This table includes participants whose first recorded therapeutic change of a biologic due to lack of effect is a discontinuation, not a dose change. Only the first such change is analyzed. Participants who had a surgery within 12 weeks prior to this change are excluded. | | | | |  |
